# Supplementary material for: O-GlcNAcylation of SIRT1 enhances its deacetylase activity and promotes cytoprotection under stress
Source: Nat Commun. 2017 Nov 14;8:1491. doi: 10.1038/s41467-017-01654-6 (PMC5684413; doi:10.1038/s41467-017-01654-6)
Supplement: Supplementary file 1 — Supplementary Information [file 41467_2017_1654_MOESM1_ESM.docx]

**

**

**Supplementary Figure. 1** The purity of the immunoprecipitated SIRT1. NCI-H1299 cells were lysed and denatured by 1% SDS lysis buffer and then diluted 10-fold with NP40 lysis buffer. SIRT1 was immunoprecipitated with antibody against SIRT1 and detected by IB using antibodies against SIRT1, OGT and DBC1. Data represent two independent experiments.





**Supplementary Figure.** 2 SIRT1 is O-GlcNAcylated at Ser549. **a** O-GlcNAcylated SIRT1 was produced by co-expressing of SIRT1 with OGT in *E. coli*. SIRT1 proteins were purified and detected by Coomassie blue staining and IB, and O-GlcNAcylation of SIRT1 was detected by IB using an anti-O-GlcNAc antibody (RL2). **b** The Q-TOF spectrum and the sequencing results of a GlcNAc-modified peptide (544–561) of SIRT1 were observed at [M+3H]^3+^ m/z 682.50. **c** Sequence alignment of the peptide containing the S549 site of human SIRT1 and the corresponding region from other mammalian SIRT1 proteins. The consensus sites are shown in shadow.





**Supplementary Figure. 3** Both wtSIRT1 and SIRT1^S549A^ effectively bind to OGT *in vitro*. Either recombinant His tagged wtSIRT1 or SIRT1^S549A^ was incubated with GST or GST tagged OGT in *vitro*. GST pulldown assays were carried out followed by immunoblotting with anti-GST and anti-SIRT1 antibodies. Data represent two independent experiments.





**Supplementary Figure. 4** The shSIRT1 efficiently silences endogenous but not exogenous SIRT1. NCI-H1299 cells were transfected with the expression vector of shRNA targeting the 3’-UTR of SIRT1 mRNA combined with/without SIRT1 expression vector, and the expression of SIRT1 was detected by IB with anti-SIRT1 antibody. Data represent three independent experiments.





**Supplementary Figure. 5** The relative deacetylase activity of the *in vitro* O-GlcNAcylated SIRT1. **a** The wtSIRT1 or SIRT1^S549A^ were co-expressed with or without OGT in *E. coli*, and the purified SIRT1 proteins were analysed by SDS–PAGE and their O-GlcNAcylation levels were detected by IB with RL2 antibody, respectively. Data represent two independent experiments. **b** The relative deacetylase activity was determined *in vitro* at the concentration of 25 μM fluorogenic acetylated p53 peptide substrate and 500 μM NAD^+^ using a fluorometric assay system. Data represent two independent experiments. Student’s *t*-test. Results are expressed as the mean ± s.d. (n = 3 biologic replicates). *P* values: ***P*<0.01, ****P*<0.001.


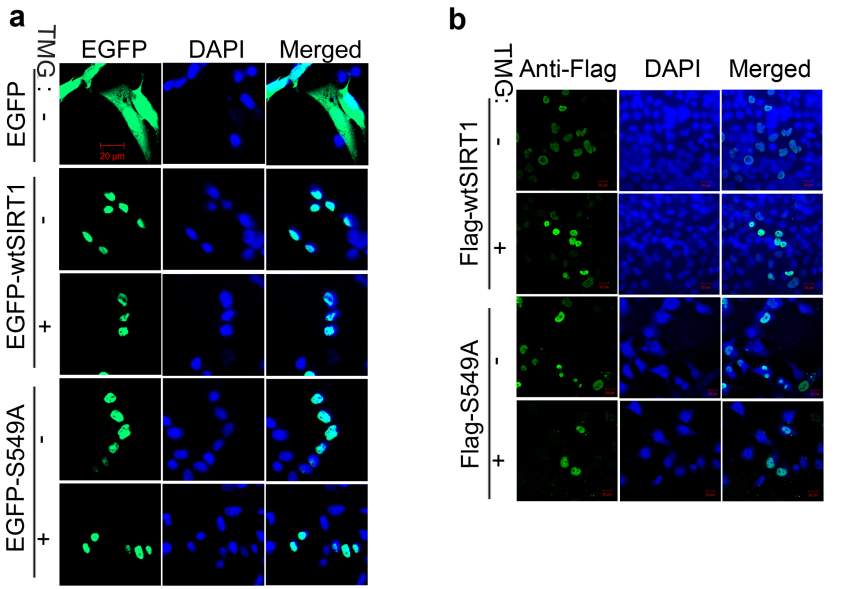


**Supplementary Figure. 6** O-GlcNAcylation doesn’t affect the cellular distribution of SIRT1. **a** NCI-H1299 cells were transfected with EGFP-fused wtSIRT1 or SIRT1^S549A^, and either in the presence of 2 μM TMG for 4 h or untreated. The cellular distribution of SIRT1 was determined by confocal imaging. **b** NCI-H1299 cells were transfected with Flag-tagged wtSIRT1 or SIRT1^S549A^, which were then in the presence or absence of 2 μM TMG for 4 h. The cellular distribution of SIRT1 was determined by fluorescence staining with anti-Flag antibody and confocal imaging. DAPI staining was used to identify the nucleus. Scale bars: 20 μm.





**Supplementary Figure. 7** Stress stimuli enhance the O-GlcNAcylation of SIRT1. HCT 116 cells were transfected with Flag-tagged wtSIRT1 or SIRT1^S549A^. Chemoenzymatic labelling and IB analysis were used to detect SIRT1 O-GlcNAcylation from untreated cells, as well as cells treated with 25 μM etoposide, 200 μM H_2_O_2_ or glucose depletion (fasting). Data represent two independent experiments.


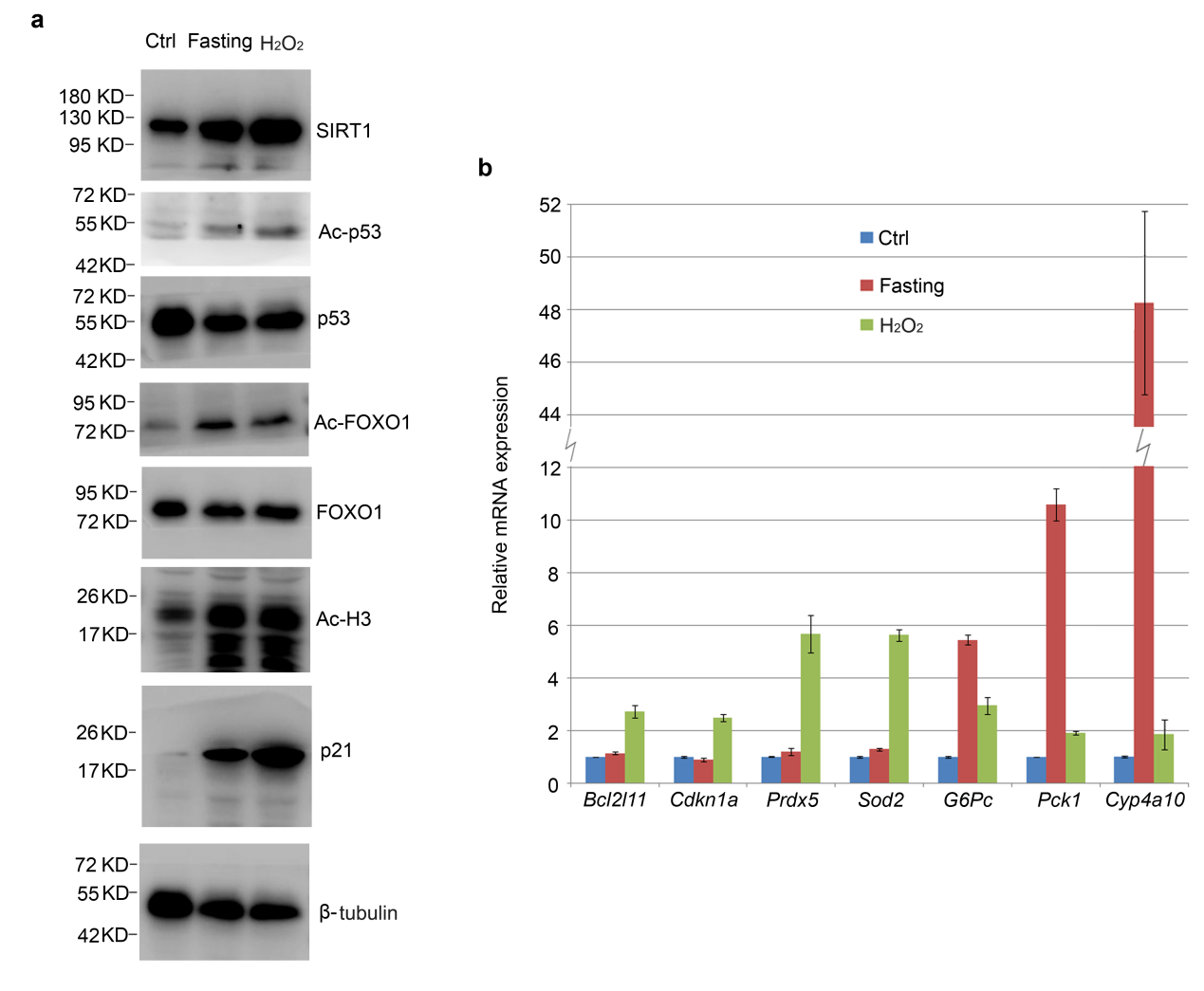
**Supplementary Figure. 8** Stress stimuli affect protein acetylation and expression in mouse liver. Balb/c mice were either fasted for 18 h or injected intraperitoneally with 200 μl of 100 mM H_2_O_2_. **a** Whole liver extracts were immunoblotted with the indicated antibodies. Data represent two independent experiments. **b** Total RNA was extracted from mouse livers and reverse-transcribed. Real-time PCR were used to detect the mRNA expression of the indicated genes. Six mice per group were used and samples from two mice were mixed in equal amounts for Real-time PCR detection, representative data from two independent experiments. Results are expressed as the mean ± s.d. (n = 3 samples per group).





**Supplementary Figure. 9** O-GlcNAcylation of SIRT1 inhibits the transcriptional activity of p53. **a** NCI-H1299 cells were transiently transfected with a p53-activated luciferase reporter construct and p53, in combination with or without either wtSIRT1 or SIRT1^S549A^ as indicated. The cells were in the presence or absence of 25 μM etoposide for 24 h, and the luciferase activities were assayed. **b** Flag-tagged wtSIRT1 or SIRT1^S549A^ was co-expressed with or without p53 in NCI-H1299 cells, which were treated with either 25 μM etoposide or solvent for 24 h. Then, the mRNA levels of *CDKN1A* were measured using real-time PCR. Student’s *t*-test. Results are expressed as the mean ± s.d. (n = 3 biologic replicates). *P* values: ****P*<0.001.





**Supplementary Figure. 10** The stable silencing and re-expression of SIRT1 in NCI-H1299 cells. NCI-H1299 cells were infected with lentiviruses expressing shRNA targeting the 3’-UTR of SIRT1 combined with or without retroviruses expressing wtSIRT1 or SIRT1^S549A^, which were then selected with puromycin and hygromycin B. The expression of SIRT1 and β-tubulin were detected by IB with antibodies against SIRT1 and β-tubulin. Data represent three independent experiments.

**
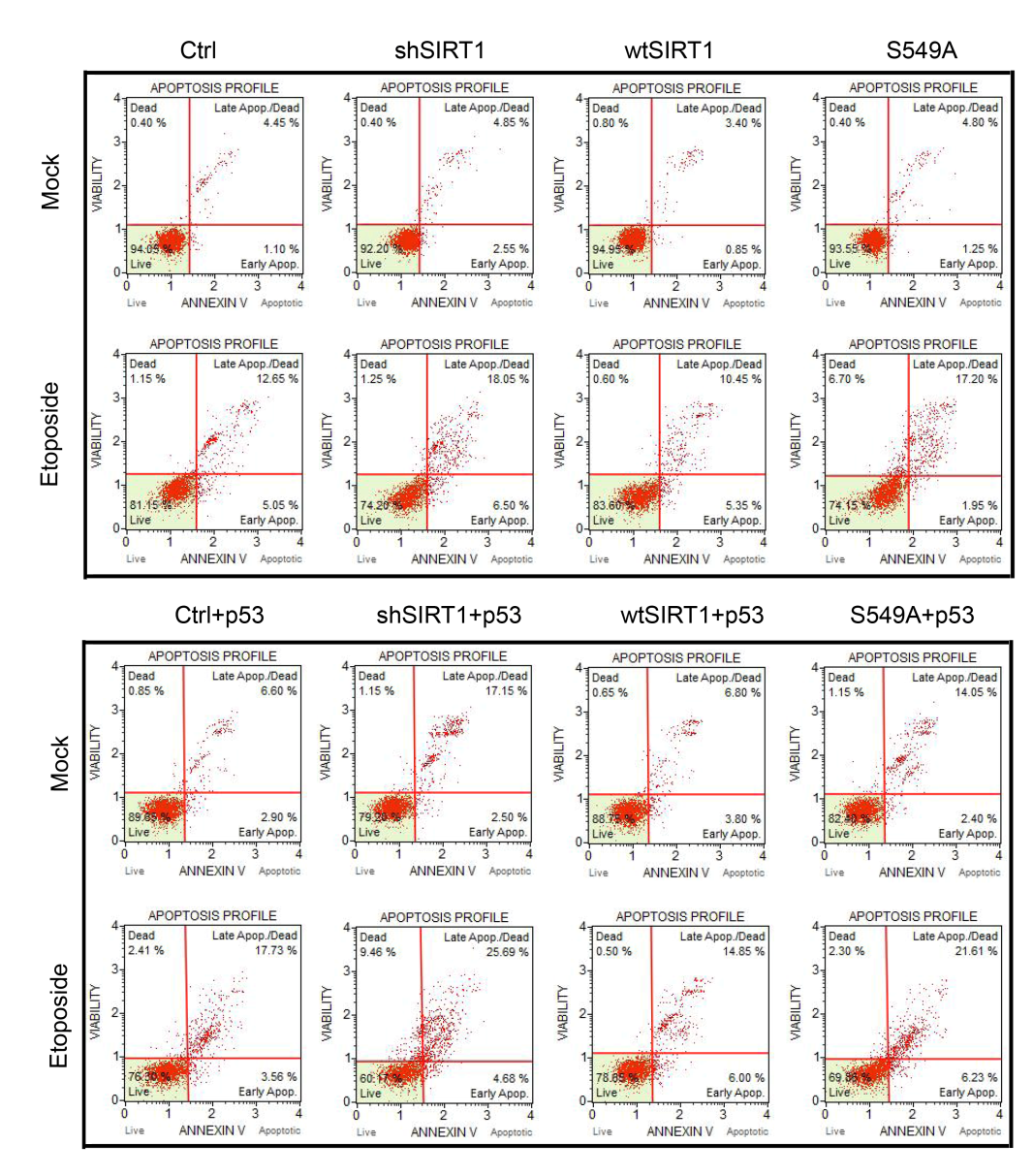
**

**Supplementary Figure. 11** The representative fluorescence intensity scatter plots analysed by Muse Annexin V & Dead Cell Kit on Muse Cell Analyzer. Data represent three independent experiments.





**Supplementary Figure. 12** O-GlcNAcylation of SIRT1 protects cells from death. NCI-H1299 cells, which were stably silenced endogenous SIRT1 and expressed wtSIRT1 or SIRT1^S549A^, were transfected with/without p53 and treated with etoposide or solvent for 24 h. Then, the activated caspases-3 and cleaved PARP proteins were detected by IB **a** and the activity of caspase-3/-7 was assayed **b**. Data represent two independent experiments. Student’s *t*-test. Results are expressed as the mean ± s.d. (n = 3 biologic replicates). *P* values: **P*<0.05, ***P*<0.01, ****P*<0.001.


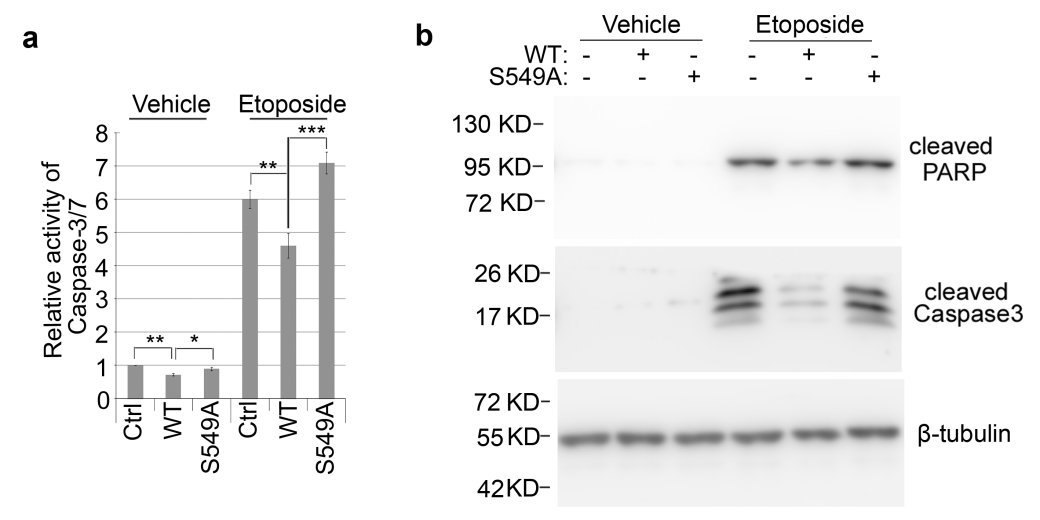


**Supplementary Figure. 13** O-GlcNAcylation of SIRT1 protects HCT 116 cells from apoptosis by deacetylating p53 upon exposure to stress stimuli. HCT 116 cells transfected with Flag-tagged wtSIRT1 or SIRT1^S549A^ were treated with either 25 μM etoposide or solvent for 24 h. Data represent two independent experiments. **a** Activities of caspase-3/7 were assayed. **b** The activated caspases-3 and cleaved PARP were detected by IB. Data represent 1 out of 3 experiments. Data represent two independent experiments. Student’s *t*-test. Results are expressed as the mean ± s.d. (n = 3 biologic replicates). *P* values: **P*<0.05, ***P*<0.01, ****P*<0.001.


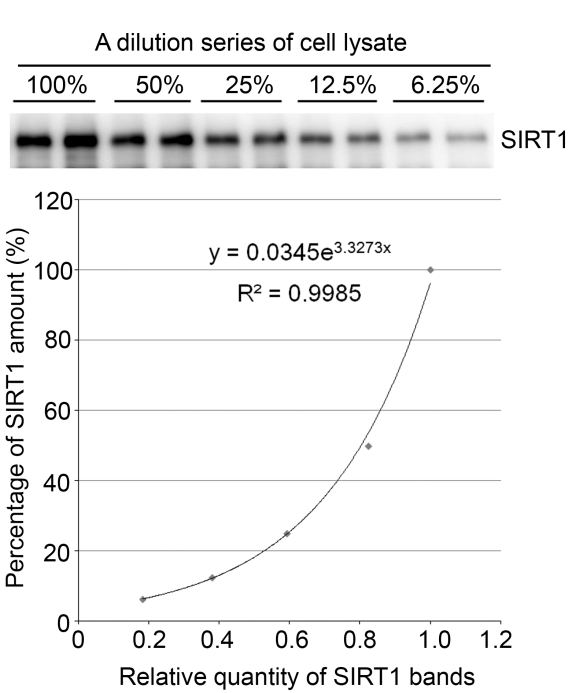


**Supplementary Figure. 14** The standard curve for SIRT1 relative quantification by IB. **a** A dilution series of NCI-H1299 cell lysate were immunoblotted with anti-SIRT1 antibody. **b** The relative intensity of every SIRT1 band was quantified using the GE IQTL analysis software. The standard curve and the curve equation were obtained. Data represent two independent experiments.





**Supplementary Figure. 15** Original data of immunoblots for Fig. 1.





**Supplementary Figure. 16** Original data of immunoblots for Fig. 2.

**
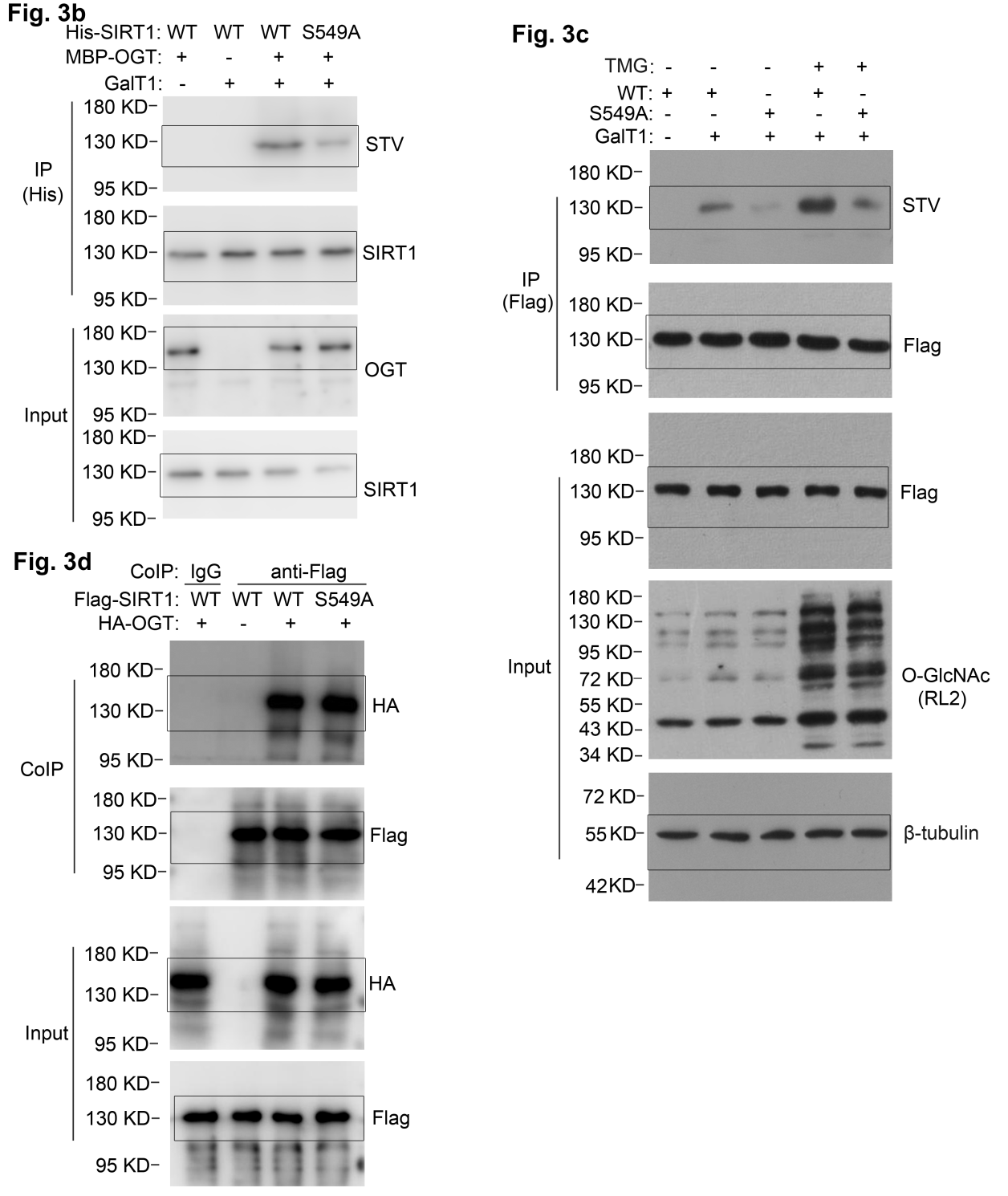
**

**Supplementary Figure. 17** Original data of immunoblots for Fig. 3.

**

**

**Supplementary Figure. 18** Original data of immunoblots for Fig. 4.

**

**

**Supplementary Figure. 19** Original data of immunoblots for Fig. 5.

**

**

**Supplementary Figure. 20** Original data of immunoblots for Fig. 6.

| **SIRT1** | **Km**  (μM) | **Kcat**  (min^-1^) | **Kcat/Km**  (min^-1^ M^-1^) |
| --- | --- | --- | --- |
| WT-GlcNAc | 61.8±2.9 | 2.18±0.075 | 35280 |
| WT | 88.7±3.6 | 1.42±0.036 | 15967 |
| S549A-GlcNAc | 50.5±5.0 | 2.32±0.041 | 45978 |
| S549A | 60.3±4.2 | 1.61±0.062 | 26682 |

**Supplementary Table 1 The enzyme kinetic parameters.** The enzyme kinetic parameters were detected by varying the p53 (K382Ac) fluorometric peptide substrate concentration while keeping NAD^+^ at a saturating concentration for wtSIRT1 (WT), wtSIRT1 bearing O-GlcNAc (WT-GlcNAc), SIRT1^S549A^ (S549A), and SIRT1^S549A^ bearing O-GlcNAc (S549A-GlcNAc). The enzyme kinetic parameters were showed. Results are expressed as the mean ± s.d. (n=3 independent experiments).

| **Gene name** | **Forward primer** | **Reverse primer** |
| --- | --- | --- |
| Human *CDKN1A* | 5’-CCTGTCACTGTCTTGTACCCTTG-3’ | 5’-GAAATCTGTCATGCTGGTCTGC-3’ |
| Human *BBC3* | 5’-TCAGCCCTCGCTCTCGC-3’ | 5’-TCGTCCGCCATCCGC-3’ |
| Human *GAPDH* | 5’-GGGAAGGTGAAGGTCGGAGT-3’ | 5’-TGTAGTTGAGGTCAATGAAGGGG-3’ |
| Mouse *Cdkn1a* | 5’-ACAGGCACCATGTCCAATCCT-3’ | 5’-CGAAGTCAAAGTTCCACCGTTCT |
| Mouse *Bcl2l11* | 5’-CCCTACAGACAGAACCGCAAGA-3’ | 5’-TACCAGGCATCACCGTGGATA-3’ |
| Mouse *Sod2* | 5’-GAACAATCTCAACGCCACCG-3’ | 5’-GCTTGATAGCCTCCAGCAACTCT-3’ |
| Mouse *Prdx5* | 5’-GAGCCCGCAGCTTCAGCA-3’ | 5’-GCCCCAGGGACTCCAAACA-3’ |
| Mouse *Pck1* | 5’-TGGATGTCGGAAGAGGACTTTG-3’ | 5’-AGGGCGAGTCTGTCAGTTCAATAC-3’ |
| Mouse *G6Pc* | 5’-TCCTCTGGGTGGCAGTGGTC-3’ | 5’-CGGAGCTGTTGCTGTAGTAGTCG-3’ |
| Mouse *Cyp4a10* | 5’-AACTTCCCAAGTGCCTTTCCTAG-3’ | 5’-AACAAGAGCAAACCATACCCAATC-3’ |
| Mouse *Gapdh* | 5’-AATTCAACGGCACAGTCAAGG-3’ | 5’-TGTTAGTGGGGTCTCGCTCC-3’ |

**Supplementary table 2: List of primers.** Primer sequences used for RT-qPCR analysis in mouse and human samples.
